# Most lobular carcinoma in situ and atypical lobular hyperplasia diagnosed on core needle biopsy can be managed clinically with radiologic follow-up in a multidisciplinary setting

**DOI:** 10.1002/cam4.223

**Published:** 2014-03-18

**Authors:** Lavinia P Middleton, Nour Sneige, Robin Coyne, Yu Shen, Wenli Dong, Peter Dempsey, Therese B Bevers

**Affiliations:** 1 Department of Pathology, The University of Texas MD Anderson Cancer CenterHouston, Texas; 2 Department of Clinical Cancer Prevention, The University of Texas MD Anderson Cancer CenterHouston, Texas; 3 Department of Biostatistics, The University of Texas MD Anderson Cancer CenterHouston, Texas; 4 Department of Diagnostic Radiology, Section of Breast Imaging, The University of Texas MD Anderson Cancer CenterHouston, Texas

**Keywords:** Atypical lobular hyperplasia, biopsy, lobular carcinoma in situ, lobular neoplasia, upgrade

## Abstract

We evaluated the efficacy of using standard radiologic and histologic criteria to guide the follow-up of patients with lobular carcinoma in situ (LCIS), lobular neoplasia (LN), or atypical lobular hyperplasia (ALH). Patients with high-risk benign lesions diagnosed on biopsy were presented and reviewed in a multidisciplinary clinical management conference from 1 November 2003 through September 2011. Associations between patient characteristics and rates of upgrade were determined by univariate and multivariate logistic models, and times to diagnosis carcinoma were calculated. Of 853 cases reviewed, 124 (14.5%) were lobular neoplasms. In all, 104 patients were clinically and/or radiographically monitored. In 20 patients, who were found to have LN on core biopsy and were recommended to have immediate surgical excision, a more significant lesion was identified in 8 (40%) of the excised specimens. Factors associated with a more significant lesion on excisional biopsy included whether the lobular lesion had been targeted for biopsy and whether the extent of disease involved three or more terminal duct lobular units. Of the 104 patients radiographically and clinically monitored, the median follow-up time was 3.4 years with a range of 0.44–8.6 years. Five patients under surveillance were subsequently diagnosed with breast malignancy (three of the five at a site unrelated to the initial biopsy). Patients with incidental lobular lesions identified on percutaneous core needle biopsy have a small risk of upgrade and may not require an excisional biopsy. Clinical management of low-volume lobular lesions in a multidisciplinary setting is an efficacious alternative to surgical excision when radiologic and histologic characteristics are well-defined.

## Introduction

Atypical lobular hyperplasia (ALH) and lobular carcinoma in situ (LCIS) are both risk factors for and nonobligate precursors of invasive carcinoma 1–3. LCIS was first illustrated by Ewing in 1919 4 and defined in 1941 by Foote and Stewart 5 as an in situ lesion associated with invasive lobular carcinoma, however, the management of LCIS was previously not considered relevant prior to the 1980s as treatment was targeted to address only the associated invasive carcinoma.

Currently, in the era of screening mammography, LCIS and ALH are not uncommonly identified as the most significant pathology in biopsies performed for breast abnormalities and their management remains controversial. Additionally, with more women being screened, and more sensitive imaging techniques being used than in the past, the incidence of finding a lobular lesion on core biopsy is increasing 6–8.

The risk of the development of an ipsilateral or contralateral breast cancer is reported to be 4–5 times higher in patients with ALH and 8–10 times higher in patients with LCIS than in the general population 9–11. Epidemiologic studies have shown that 10–20% of patients diagnosed with LCIS will subsequently develop breast carcinoma 15–25 years after their initial diagnosis 12–15. Recent studies have also shown that the risk of developing a subsequent invasive carcinoma after the diagnosis of ALH and/or LCIS is three times more likely to occur in the ipsilateral breast than in the contralateral breast 14 and patients with ALH or LCIS have an overrepresentation of developing invasive lobular carcinoma when compared with the general population 13,16,17.

Our challenge as clinicians is to correctly triage which patients need immediate surgery and which patients can be treated with chemoprevention and monitored by imaging. It is the practice at our institution to review all patients diagnosed with benign, high-risk lesions in a multidisciplinary management conference that includes representation from the disciplines of pathology, radiology, cancer prevention, and surgery. The goal of these conferences is to distinguish which patients would be best served by clinical management comprising chemoprevention and clinical and radiologic surveillance and which patients will likely need an excisional biopsy to rule out coexisting higher-grade lesions. Rigorous conference preparation is necessary with imaging and pathology studies rereviewed according to set guidelines. During the conference clinicians arrive at a consensus final disposition for each patient and all pertinent data are recorded (Table 1). Some patients are dispositioned to receive chemoprevention consisting of either tamoxifen citrate (20 mg daily for 5 years) or raloxifene (60 mg daily for 5 years) with radiologic follow-up at regular intervals. Alternatively, surgical excision is recommended for patients based on radiologic determination of residual calcifications and histologic assessment of association of lobular neoplasia (LN) with targeted lesions as opposed to when LN is incidental to calcifications. The purpose of this study was to identify radiographic and pathologic criteria that suggest an increased likelihood of upgrade at the time of surgical excision in patients with ALH/LN or LCIS and conversely, to identify pathologic and radiographic features in a subset of patients with very low risk of an upgrade who could be monitored. To do this, we retrospectively reviewed records of patients at high risk of cancer who were found to have ALH, LCIS, or LN on core needle biopsy.

**Table 1:** Clinical management conference checklist used to evaluate each patient presented at multidisciplinary conference prior to disposition

Diagnosis	Clinical abnormality	Radiologic abnormality	Biopsy type
ALH	**Mass—ass c/imaging abn—No**	**Architectual distortion**	Stereo, <half excised
ADH	**Mass—ass c/imaging abn—Yes**	**Asymmetry**	Stereo, >half excised
PASH	**Thickening—ass c/imaging abn—No**	**Calcifications**	Stereo, >90% excised
Other	**Thickening—ass c/imaging abn—Yes**	**Enhancing lesion**	US FNA
Papillary lesion	**Skin change—ass c/imaging abn—No**	**Mass**	US core—lesion excised—no
Papillomitosis	**Skin change—ass c/imaging abn—Yes**	**Filling defect—ductogram**	US core—lesion excised—yes
Juvenile papilloma	**N. discharge ass c/imaging abn—No**	**None**	US core vac—lesion excised—no
PASH	**N. discharge—ass c/imaging abn—Yes**		US core vac—lesion excised—yes
Fibroepithelial lesion	**N. retraction—ass c/imaging abn—No**	Location (radiologic abn):	MRI core—lesion excised—no
Fibroadenoma	**N. retraction—ass c/imaging abn—Yes**	___o'clock **Subareolar**	MRI core—lesion excised—yes
ALH—focal (<3 lobules)—targeted	**None**		Outside biopsy
ALH—focal (<3 lobules)—incidental	Location (papable) breast		**Number of cores:/cals**
ALH—extensive (>2 lobules)—targeted	**___0'clock position**		________
ALH—extensive (>2 lobules)—incidental	**Right Left**		
ADH—focal (<3 lobules)—targeted	**Bilateral Subareolar**		
ADH—focal (<3 lobules)—incidental	**Not applicable**		
ADH—extensive (>2 lobules)—targeted			
ADH—extensive (>2 lobules)—incidental			
Classic LCIS			
P. LCIS—focal (<3 lobules)—targeted			
P. LCIS—focal (<3 lobules)—incidental			
P. LCIS—extensive (>2 lobules)—targeted			
P. LCIS—extensive (>2 lobules)—incidental			
Papilloma with atypia		Mammogram BIRADS:	
Papilloma		**Zero**	
Radial scar		**1**	
Columnar cell change	Disposition	**2**	
Flat epithelial atypia, focal, T	**Annual F/U**	**3**	**Needle gauge:__**
Flat epithelial atypia, focal, I	**MMG—6 months F/U**	**4**	
Flat epithelial atypia, extensive, T	**US—6 months F/U**	**4A**	
Flat epithelial atypia, extensive, I	**MRI—6 months F/U**	**4B**	
Flat epithelial atypia, CAPSS c/atypia	**MMG/MRI—6 months F/U**	**4C**	
Benign	**MMG/US—6 months F/U**	**5**	
	**CBE—6 months F/U**	BREAST:	
	**Further biopsy**	**Left**	
	**Surgical excision**	**Right**	
	**Review prior pathology/imaging**	Bilateral	

Abn, abnormality; ADH, atypical ductal hyperplasia; ALH, atypical lobular hyperplasia; ass, associated; CBE, clinical breast exam; F/U, follow-up; I, Incidental; LCIS, lobular carcinoma in situ; MMG, mammogram; MRI, magnetic resonance imaging; N, nipple; PASH, psuedoangiomatous stromal invasion; P. LCIS, pleomorphic lobular carcinoma in situ; Stereo, stereotactic guided; T, Targeted; US, ultrasound.

## Materials and Methods

**Figure 1:**
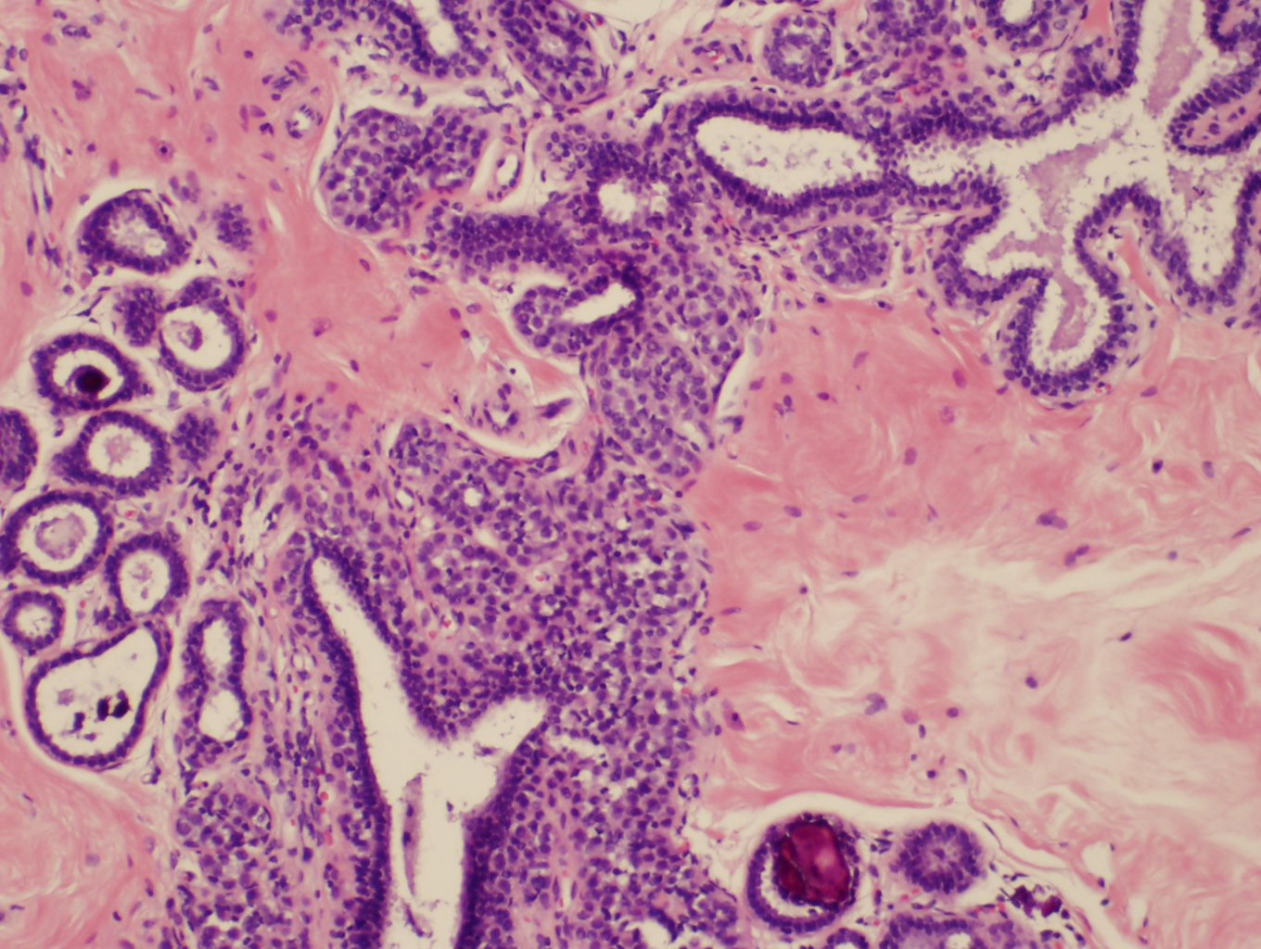
Photomicrograph of atypical lobular hyperplasia associated with columnar cell changes and incidental to targeted microcalcifications.

We reviewed records of 853 patients from our institution's multidisciplinary clinical management conferences from November 2003 through September 2011 and identified 124 patients with the dominant diagnosis of LN, ALH, or LCIS on core biopsy. The confidentiality of the patient's health record was maintained in accordance with HIPPA. This project was approved by internal IRB (Protocol PA12-0194). The diagnosis of LCIS was based on a monotonous discohesive proliferation of cells occupying the terminal ductal lobular units (TDLUs) and ducts. According to the criteria proposed by Page, LCIS was diagnosed if 50% or more of a TDLU's ducts were involved, and ALH was diagnosed if the discohesive monotonous proliferation of cells occupied less than 50% of a TDLU or if only pagetoid extension a duct by these cells was noted 13 (Fig. 1). LN, is a term introduced by Haagensen in 1978 to describe lobular proliferations involving preexisting lesions like adenosis and includes both ALH and LCIS in the spectrum 18. In our study, the diagnosis of LN was most often reserved for low nuclear grade, monotonous, discohesive epithelial proliferations involving sclerosing adenosis.

ALH, LCIS, or LN was the most significant lesion identified in the cases included in this analysis, and biopsies containing coexisting atypical ductal hyperplasia, ductal carcinoma in situ (DCIS), or invasive carcinoma were excluded from review. Cases of ALH, LCIS, or LN with coexisting radial scar and papillomas were also excluded as these patients were evaluated for immediate excision. Patients with either pleomorphic LCIS, defined as LCIS with grade 2 or 3 nuclei, or florid LCIS with necrosis also were excluded from this study, as all patients in our institution diagnosed with variant LCIS are recommended to have excisional biopsies with negative margins 19,20. Routinely, two hematoxylin and eosin–stained slides were reviewed from each core biopsy obtained.

All cases were reviewed by a dedicated breast pathologist and a breast radiologist. Variables that were considered included whether the ALH, LCIS, or LN was focal (defined as ≤2 TDLU involvement) or extensive (defined as ≥3 TDLU involvement), whether the ALH, LCIS, or LN was associated with the targeted lesion or incidental, the percentage of the lesion sampled (categorized as either <50% or 50–90% of calcifications excised by stereotactic biopsy) or status of lesion excision on ultrasound or MRI-guided biopsy, and the concordance of histopathologic and radiologic findings. Radiographic images of patients triaged to clinical follow-up and subsequently diagnosed with breast cancer were reviewed to ascertain correlation between the location of the ALH, LCIS, or LN found on biopsy and that of the ensuing breast cancer.

### Biopsy methods

Biopsies were performed for radiographic findings of calcifications in 89 (71.8%) patients, masses in 17 (13.7%) patients, and masses and calcifications in 3 (2.4%) patients. In the remaining (12.1%) patients, biopsies were performed for architectural enhancing lesions in nine patients, architectural distortion in four, and asymmetry in two. The biopsy mode was stereotactic in 70 (56.5%) patients, ultrasound-guided core in 9 (7.3%), magnetic resonance image (MRI)-guided in 7 (5.6%), and unknown in 38 (30.6%) patients whose biopsy procedures were performed at other institutions. The number of cores per patient obtained ranged from 1 to 20, with the majority of cases (63.3%) falling into the range of 6–10 cores per patient. Of the patients whose biopsies were performed for calcifications at our institution, greater than 50% of the calcifications were removed in 46 (50.0%) patients, less than 50% of the calcifications were removed in 29 (31.5%). The needle gauge was 9 (80%) in 72 biopsies, 10 (1.1%) in 1, 11 (7.8%) in 7, 12 (3.3%) in 3, 14 (1.1%) in 1, and 16 (6.7%) in 6. The needle gauge was not identifiable in 34 of the samples obtained outside our institution.

### Statistical methods

Descriptive statistics were used to summarize the data. Categorical variables were described by frequencies and percentages. The Fisher exact test was used to evaluate the association between two categorical variables. Uni- and multicovariate logistic models were used to investigate the effects of radiographic and pathologic characteristics on the recommendation for immediate surgical excision and on lesion upgrade. Patients' data were censored at the last follow-up if a diagnosis of invasive breast cancer or DCIS did not occur on or before the last follow-up. For the patients who were not recommended for surgical excision, the upgrade events in the patient population were modeled using Poisson distribution. The incidence rate was estimated as the number of upgraded cases observed divided by the cumulative time at risk of upgrade during the observation period, and its 95% exact Poisson confidence limits (CL) for the incidence rate were estimated. All tests were two sided, and *P-*values less than 0.05 were considered statistically significant. All analyses were conducted using SAS (version 9.2, Cary, NC) and S-plus (version 8.0, TIBCO Software Inc., Palo Alto, CA) statistical software.

## Results

### Radiographic and pathologic characteristics

In total, 124 cases of ALH, LCIS, or LN were detected on core biopsy. Among these, there were 77 (62.1%) cases of ALH alone, 33 (26.6%) cases of LCIS, and 14 (11.3%) cases of LN. Thirty-nine (31.5%) lesions were extensive (involving ≥3 TDLU) and 85 (68.5%) were focal (involving ≤2 TDLU); 82 (66.1%) lesions were found incidentally, and 42 (33.9%) were targeted. The amount of the lesion removed was associated with needle gauge (*P* < 0.001).

### Radiographic and pathologic characteristics by type of diagnosis

Calcifications were the most common radiographic finding, occurring in 77.9% of ALH cases, 60.6% of LCIS cases, and 64.3% of LN cases. Patients diagnosed with LCIS were more likely to be recommended for immediate surgical excision of their lesions than were patients diagnosed with ALH or LN (LCIS, 36.4%; LN, 21.4%; ALH, 6.5%; *P* = 0.004). LCIS cases were more likely to be upgraded to invasive cancer or DCIS (LCIS, 23.8%; ALH, 1.7% *P* = 0.003).

### Radiographic and pathologic characteristics of lesions recommended for immediate excision secondary to radiology and pathology discordance

A subset of patients with ALH/LCIS/LN was recommended to undergo immediate excision due to discordance between radiologic findings and pathology results. Twenty (16.1%) patients were recommended to undergo immediate surgical excision during the multidisciplinary conference. The cases with extensive lesions or targeted lesions were more likely to be recommended for surgical excision than were those with focal or incidental lesions (*P* < 0.001). Cases diagnosed as ALH were less likely than those diagnosed as LCIS or LN—and cases with calcification only were less likely than those with masses or other radiologic findings—to be recommended for surgical excision (*P* < 0.01). Cases with five or fewer biopsy cores were more likely than those with six or more cores to be recommended for surgical excision (*P* = 0.024). In unicovariate logistic analysis, LCIS that was extensive, targeted, and associated with mass lesions were statistically significantly associated with the recommendation for immediate surgical excision (*P* < 0.05). In the multicovariate logistic model, extensive versus focal lesions (odds ratio [OR], 10.68; 95% confidence interval [CI], 3.33–34.26; *P* < 0.001) and findings of a mass or other type of abnormality versus calcification only (OR, 4.51; 95% CI, 1.46–13.91; *P* = 0.009) were associated with the recommendation for surgical excision. In 8 of the 20 cases recommended for immediate surgical excision, a more significant lesion was identified after review of the final pathology. An escalation in diagnosis was more common in cases initially diagnosed as LCIS (22.6%) than for those diagnosed as ALH (1.7%; *P* = 0.002), for extensive lesions (32.0%) than for focal lesions (0%; *P* < 0.001), and for targeted lesions (25.9%) than for incidental lesions (1.6%; *P* < 0.001). An escalation in diagnosis was less common for cases with calcification only (3.3%) than for cases with a mass or other type of abnormality (21.4%; *P* = 0.011). In all 20 cases, the excised specimen contained the original biopsy tract and the original ALH/LCIS/LN associated with the additional pathology. Since type of abnormality was significantly associated with targeted/incidental status (*P* < 0.001), type of abnormality was not significantly associated with lesion upgrade in the multicovariate model after adjusting for whether the lesion was targeted or incidental. Targeted/incidental status and type of diagnosis were significantly or marginally independently associated with lesion upgrade in the multicovariate model (Table 2).

**Table 2:** Data showing that limited volume ALH/LN/LCIS that is focal or incidental to the biopsy and adequately sampled is not likely to have a more significant upgrade on excision

Variable	Levels	Upgrade		*P* value

		Yes (*n* = 8)	No (*n* = 81)	
Focal (<3 TDLU) vs. extensive ≥3 TDLU)	Extensive	8 (32%)	17 (68%)	<0.0001
	Focal		64 (100%)	
Targeted vs. incidental	Incidental	1 (1.6%)	61 (98.4%)	0.0008
	Targeted	7 (25.9%)	20 (74.1%)	
Percentage of lesion removed during biopsy	<Half excised		17 (100%)	1
	>Half excised	2 (5.6%)	34 (94.4%)	

TDLU,Terminal Duct Lobular Unit.

### Radiographic and pathologic characteristics of patients with limited volume ALH/LCIS/LN followed clinically

Among 104 patients recommended to undergo chemoprevention and surveillance rather than immediate surgical excision the median follow-up time for radiologic surveillance was 3.4 years with a range of 0.44–8.6 years. Five patients for whom surgical excision was not recommended at the initial management conference subsequently were diagnosed with invasive cancer or DCIS during clinical follow-up. Two patients receiving semiannual mammographic follow-up were diagnosed with malignancy within 2 years; both had DCIS develop in the ipsilateral breast but in a different quadrant than the initially diagnosed ALH. One patient, with a history of right breast invasive ductal carcinoma and right breast LCIS, received chemoprophylaxis for her newly diagnosed left breast LCIS. Five years later she developed a left breast carcinoma in a different quadrant than her biopsy containing LCIS. Only 2 (1.92%) of 104 patients for whom chemoprevention and surveillance was recommended had an upgrade in the same area of the breast where the LN was identified. For these two patients, the time intervals between the disposition conference and cancer diagnosis were 38 and 66 months, respectively (Table 3).

**Table 3:** Radiology and pathology findings in 5 of 104 patients who developed carcinoma during surveillance and chemoprevention

Imaging target	CNB finding	Pathology on excision	Upgrade? Why or Why not	Interval time
Ca^++^	ALH bordering on LCIS	DCIS	True upgrade in same area as biopsy	66 mo
Ca^++^	LCIS	ILC	True upgrade in same area as biopsy	38 mo
Ca^++^	Incidental ALH	IDC	Not an upgrade; cancer developed in different area than biopsy	24 mo
Ca^++^	Incidental ALH	DCIS	Not an upgrade; patient had prior biopsy of ductal epithelial atypia. Cancer developed at site of ADH	17 mo
Ca^++^	LCIS	IDC	Not an upgrade; cancer developed in different area than biopsy	60 mo

ALH, atypical lobular hyperplasia; Ca^++^, calcifications; DCIS, ductal carcinoma in situ; IDC, invasive ductal carcinoma; ILC, invasive lobular carcinoma; LCIS, lobular carcinoma in situ; mo, months.

## Discussion

Our results show that clinical management of LCIS and ALH can be an effective alternative to surgical excision when radiologic and histologic characteristics are well-defined and suggest a low potential for an upgrade at time of excision. We recommend clinical management with serial imaging for patients having limited volume (<3 TDLUs involved by ALH, LCIS, or LN) as we found that 98% of such patients presented in a multidisciplinary clinical management conference and triaged to surveillance based on imaging, pathologic, and clinical findings will not have an upgraded lesion on excisional biopsy. This is a significant finding in a population of patients, who, because of their diagnosis, already at an increased risk of breast cancer in the ipsilateral and contralateral breast. To be clear, we recommend excision for patients diagnosed with lobular lesions associated with a mass lesion or other radiology and pathology discordance.

Patients who are found to have incidental ALH, LCIS, or LN as the most important pathologic finding on a core needle biopsy obtained for radiographic findings other than a mass lesion presents the clinician with a significant dilemma, as controversy surrounds the management of these lesions. The current National Comprehensive Cancer Network guidelines recommend excision for patients with LCIS on core biopsy in order to rule out a more significant lesion 21. These guidelines, however, do not address the finding of ALH identified on core needle biopsy and may result in over treatment with unnecessary surgery.

The results of some studies have recommended that all patients with ALH or LCIS on core needle biopsy undergo excision of the targeted lesion, but other studies have recommended close radiologic follow-up. Prior series have shown rates of upgrade to DCIS or invasive carcinoma ranging from 1% to more than 40% of cases on excision after the diagnosis of ALH or LCIS on core biopsy 22–25. Possible explanations for this wide range of upgrade rates include differences in the volume of tissue sampled, degree of pathologic–radiologic correlation, and inclusion of other histopathology in the samples studied. Our objective was to define, through participation in clinical management conference, rigorous criterion that identifies a subset of patients with ALH and LCIS that could be followed by imaging and report our experience with patients diagnosed with this entity as the most significant finding. Again, a mass lesion with discordant biopsy pathology mandates an excision. We share our checklist of criteria used to provide recommendations to follow patients having limited volume ALH, LCIS, or LN with serial imaging.

In the largest series to date, Hwang et al. evaluated the outcomes of 333 patients with ALH and LCIS on CNB with subsequent excision 22. After excluding patients with a radiologic–pathologic discordance and nonclassic tumor morphology, the authors found an upgrade rate of 1% with a mean follow-up time of 49 months. The authors concluded, and our data support, that ALH and classic LCIS with concordant radiologic and pathologic findings can be appropriately managed with clinical and radiologic follow-up without surgery 22.

Subhawong et al. studied 56 cases of ALH diagnosed on core biopsy with paired excision 26. None of the patients with ALH had cancer on excision. The study suggested that cases diagnosed on core biopsy as minimal ALH, defined as involving less than three foci, can be managed clinically with close radiologic follow-up. A series by Shah-Kahn et al. evaluated 184 cases of ALH or LN identified over an 8-year period 27. Similar to our study, all cases were reviewed by a team of dedicated breast pathologists and diagnostic imagers for pathologic classification and radiologic concordance. Excision was performed in 55% of the cases, and 45% of the patients were observed. The authors reported that 6% of their patients developed ipsilateral breast cancer during follow-up that ranged from 6 to 212 months. The authors concluded that not all patients with LN diagnosed on core needle biopsy require surgical excision, and that patients with pure ALH, demonstrating radiologic–pathologic concordance, may be safely observed.

Rendi et al. recently studied 73 patients with ALH and 33 with LCIS and reported an upgrade rate of 4.4% when evaluating patients with LN only in the absence of atypical ductal hyperplasia 28. The upgrade rate was negligible after excluding extensive LCIS (defined as four or more foci) and cases with radiologic and pathologic discordance. The authors concluded that patients with a diagnosis of LN on biopsy of calcifications identified on routine, normal-risk mammographic screening have a small risk of upgrade and may not require excisional biopsy. A limitation to the conclusions of our study that 30% of the cases reviewed had initial biopsies performed at another hospital. However, all initial radiology was rereviewed and when significant data necessary to triage the patient was not available, the patient was reimaged and rebiopsied.

In conclusion, our study supports the growing body of evidence that details that both normal-risk and high-risk patients with mammographically detected ALH and LCIS can be managed clinically as an alternative to surgical excision. Defined radiologic and histologic characteristics rigorously reviewed in a multidisciplinary team approach of limited volume lesions involving less than 3 TDLU (adequately sampled with large bore needles) can preselect individuals, both high and normal risk who will have a low potential for an upgrade at the time of excision and who may benefit from surveillance with chemoprevention as opposed to surgical intervention.
